# TFIIB Is Only ∼9 Å Away from the 5'-End of a Trimeric RNA Primer in a Functional RNA Polymerase II Preinitiation Complex

**DOI:** 10.1371/journal.pone.0119007

**Published:** 2015-03-16

**Authors:** Matthew J. Bick, Sohail Malik, Arkady Mustaev, Seth A. Darst

**Affiliations:** 1 Laboratory of Molecular Biophysics, The Rockefeller University, New York, NY, United States of America; 2 Laboratory of Biochemistry and Molecular Biology, The Rockefeller University, New York, NY, United States of America; 3 Department of Chemistry and Environmental Sciences, New Jersey Institute of Technology, University Heights, Newark, NJ, United States of America; Institute of Genetics and Molecular and Cellular Biology, FRANCE

## Abstract

Recent X-ray crystallographic studies of Pol II in complex with the general transcription factor (GTF) IIB have begun to provide insights into the mechanism of transcription initiation. These structures have also shed light on the architecture of the transcription preinitiation complex (PIC). However, structural characterization of a functional PIC is still lacking, and even the topological arrangement of the GTFs in the Pol II complex is a matter of contention. We have extended our activity-based affinity crosslinking studies, initially developed to investigate the interaction of bacterial RNA polymerase with σ, to the eukaryotic transcription machinery. Towards that end, we sought to identify GTFs that are within the Pol II active site in a functioning PIC. We provide biochemical evidence that TFIIB is located within ∼9 Å of the −2 site of promoter DNA, where it is positioned to play a role in de novo transcription initiation.

## Introduction

Multisubunit RNA polymerases (RNAPs) display a conserved core structure across the three domains of life. While one RNAP suffices for all RNA synthesis in eubacteria and archaebacteria, eukaryotic cells harbor three distinct enzymes, RNAPs I, II, and III (Pols I, II, and III). The 12-subunit Pol II is the enzyme largely responsible for transcription of protein-encoding genes [1]. Counterparts of all five of the core bacterial RNAP subunits are found in the eukaryotic Pols I, II, and III [2]. These orthologs include the two largest subunits RPB1 and RPB2, which correspond, respectively, to the bacterial β' and β subunits [2–4], RPB3 and RPB11, which correspond to the two copies of the bacterial α subunit [5], as well as the small RPB6 subunit, which corresponds to the bacterial ω subunit [6]. Consistent with the increased complexity of the eukaryotic transcriptional machinery, Pol II has several additional subunits that do not have bacterial counterparts.

Like the bacterial core RNAP, eukaryotic Pol II is incompetent on its own for promoter-specific transcription initiation. In bacteria, a single additional factor, termed σ, directs promoter-specific initiation [7]. Structural and biochemical studies have defined distinct roles for the four conserved domains of σ that lead to promoter-dependent transcription initiation. These roles include interactions with core RNAP to form the holoenzyme, and transcription start-site (TSS) selection through interactions with conserved promoter elements [8–17].

Most intriguingly, a conserved but unstructured (loop-like) segment of σ, called conserved region 3.2 [18] snakes through the RNAP active-site channel with a portion in proximity to the active center itself [9, 10]. Previously, [11] identified this region of σ as being in proximity to the γ-phosphate of the initiating (5’) nucleotide substrate using activity based affinity crosslinking. Subsequent studies indicated σ region 3.2 (σ3.2) played an important role in formation of the first phosphodiester bond [13, 19]. Since σ3.2 sits in the path of the nascent RNA transcript, it plays an important role in abortive initiation and in the transition from the initial transcribing complex to the elongation complex [8].

Eukaryotic Pol II requires at least 6 additional general transcription factors (GTFs) in order to form the promoter-specific pre-initiation complex (PIC), which is analogous to the closed promoter complex described for prokaryotic systems [1, 20]. Although recent data suggest that additional, newly identified factors also act as GTFs in certain contexts [21], for the paradigmatic case of the adenovirus major late (Ad ML) promoter, the key GTFs include TFIIA, TFIIB, TFIID, TFIIE, TFIIF, and TFIIH. Formation of the PIC takes place in a stepwise fashion, beginning with the recognition of TATA-box DNA by the TBP subunit of TFIID [22]. Next, TFIIB is recruited to promoter DNA through direct contacts with TBP and DNA [1, 20, 22]. A preformed TFIIF-Pol II complex is added through direct binding of TFIIB to both TFIIF and Pol II [1, 20]. Finally, the addition of TFIIE and TFIIH completes PIC assembly [1, 20].

It has generally been believed that during the course of evolution, the functional roles of the various σ regions came to be distributed across these GTFs, especially the three factors that interact directly with Pol II (TFIIB, TFIIE, and TFIIF). However, the precise functional counterparts of σ in eukaryotic systems have remained unidentified. Some evidence based on limited sequence conservation [23], and structural [24, 25] and biochemical analysis pointed to TFIIF [26, 27]. Thus, the Rap30 subunit of TFIIF (mammalian TFIIF is composed of 2 subunits, Rap74 and Rap30) shows apparent sequence homology to *E*. *coli* σ70 region 2 (31%) and *B*. *subtilis* σ43 (28%) [23]. Interestingly, Rap30 is able to bind to *E*. *coli* RNAP and is displaced by σ70; conversely, σ70 binds Pol II and is dissociated by Rap30 [28]. Furthermore, TFIIF regulates the interaction of Pol II and promoter DNA by reducing the affinity of Pol II for free DNA containing either promoter or non-promoter DNA [29], which is a known function of σ. TFIIF also appears to play a role in start site selection [26, 30, 31]. However, rather than through a direct interaction with DNA, this may be due to the stabilization of IIB in the active site [27].

More recent evidence has pointed to TFIIB as possibly playing some σ-like roles during initiation. TFIIB recruits Pol II to promoter DNA through its Zn-ribbon and linker domains [32–35]. Prior to Pol II recruitment, TFIIB binds to and helps to stabilize TBP to TATA-box DNA through its C-terminal domain [22, 35], in part through recognition of specific DNA sequences flanking the TATA-box [36, 37]. TFIIB also functions at steps subsequent to Pol II recruitment. Mutations to an N-terminal ‘reader’ region of TFIIB have been shown to affect overall transcription start site selection [38–41]. Indeed, structures of Pol II in complex with TFIIB place this B-reader within the active site cleft in proximity to the active center [39, 42–44], analogous to the structural role of σ3.2. Akin to what was described for σ [9], structural and biochemical studies further revealed a role for TFIIB in “overseeing” the promoter escape step, wherein the B-reader first sterically clashes with the nascent RNA chain of approximately 13 nucleotides in length, but ultimately yields to the RNA once a stable ternary complex can be formed [42, 44, 45].

Although X-ray crystallographic studies of Pol II have begun to provide insights into the mechanism of transcription initiation, it has not yet been possible to describe structures of Pol II and Pol II with TFIIB in the context of a functional PIC. Previous Pol II-TFIIB co-crystal structures have helped to define the topological architecture of the PIC [39, 42, 43]. A more recent Pol II-TFIIB structure has further clarified IIB's role in transcription initiation, and has provided additional evidence for the IIB-σ connection [44]. Towards identifying how the various roles of σ are fulfilled by eukaryotic GTFs, we used an activity-based affinity chemical crosslinking approach to identify GTFs that are proximal to the Pol II active site in a functioning PIC. We provide biochemical evidence that TFIIB is located within ∼9 Å of the −2 site of promoter DNA where, in a manner analogous to σ70 3.2, it is positioned to play a role in de novo transcription initiation.

## Materials and Methods

### Protein Expression and Purification

Pol II, his-tagged TFIIB, TFIIE, TFIIF, and TFIIH were isolated exactly as described [46]. Flag-tagged TFIIB was purified as described [47]. His-tagged TBP was purified as described [48].

### Synthesis of Crosslinking Derivatives of the Initiating Substrate and RNA Primers

To a solution of 36mg (0.2 mmol) of 4-azido-1-fluoro-2-nitrobenzene in 0.2 ml of DMF 30 ml of ethylenediamine was added, followed by incubation of the reaction mixture for 20 min at room temperature, and an additional 20 min at 50°C. The reaction mixture was diluted by water and extracted with chloroform. The organic layer was collected, dried over anhydrous sodium sulfate, evaporated *in vacuo*, and dissolved in 0.1 ml of anhydrous DMSO. A solution of 0.5–1 mmol of triethylammonium salt of AMP, pCpA, or pUpCpA in 50 ml of DMSO was supplemented with 30 mg each of triphenylphosphine, 2,2’-dipyridyldisusphide, and 10 ml of N-methylimidazole. After a 30 min incubation at 20°C, the mixture was precipitated with 1 ml of ether, washed with ether and air-dried. The residue was dissolved in 30 ml of DMSO and supplemented with 10 ml of the solution of the ethylenediamine derivative of azidonitrobenzene (see above). After a 20 min incubation at room temperature the mixture was precipitated by ether, the residue collected by centrifugation, dissolved in water and subjected to HPLC chromatography on a C_18_ 250 x 4 mm column using a 30 ml gradient of acetonitrile in water (0–40%). The flow rate was 1 ml/min. Fractions containing the main colored products were collected and evaporated to dryness under reduced pressure. The UV absorption spectra of the products were close to a superposition of the UV spectra for the starting nucleotide compounds and the ethylenediamine derivative of azidonitrobenzene. Acidic hydrolysis of the products (pH 2, 37°C, 1 h) yielded a mixture of the starting nucleotides and the ethylenediamine derivative of azidonitrobenzene. The products exhibited characteristic spectral changes upon irradiation at 360 nm, due to photoreaction of the azido group.

### Abortive Initiation

Abortive initiation reactions were assembled on ice in eppendorf tubes and consisted of 3 μl of assay cocktail (1.25 μl of 10X assay mix (0.2 M HEPES, pH 8.2, 40 mM MgCl_2_, prepared fresh each experiment), 0.25 μl of 20 mg/ml BSA, 0.25 μl of RNasin (40 units/μl, Promega), 0.625 μl DTT, 0.25 μl 40% PEG-8000, and ddH_2_O to 3 μl), 1 μl of 50 ng/μl plasmid pTREruΔ53 containing the adenoviral major late promoter (Ad ML), 3.5 μl of a Pol II/general transcription factor (GTF) mix of GTFs and Pol II that contained 20 ng Pol II, 8 ng TBP, 8 ng TFIIB, 2 ng TFIIEα, 1.0 ng TFIIEβ, and 10 ng TFIIF in BC100 (BC100 alone was added for the “no protein” control), and 4 μl of BC100 (20 mM Tris-Cl, pH 7.4, 20% glycerol, 0.1 mM EDTA, 0.5 mM PMSF, 2 mM DTT, 100 mM KCl). This mixture was incubated on ice for 50 min at 30°C. 1 μl of a 2:1:1 nucleotide mix (10 mM of either UCA or CA crosslinking nucleotide analog, or ATP, 20 μM cold CTP, α-^32^P-CTP (3000Ci/ml) was added, the solution was mixed by gentle pipetting and incubated for an additional 30 min at 30°C. To stop the reaction, the reactions were heated to 65°C for 5 min, and then put on ice for 2 min. 1 μl of CIP (New England Biolabs), diluted to 2 units/μl in BC100, was added to the reactions in order to clear out unincorporated nucleotides, and the reactions were incubated for an additional 30 min at 30°C. The reactions were separated on a 25% PAGE gel containing 7 M urea and 1X TBE buffer. The gels were wrapped in Saran and imaged using BioMax MR film (Kodak) after an overnight incubation at −80°C.

### Crosslinking

Crosslinking experiments were set up in a similar fashion as the abortive initiation reactions. Reactions were assembled on ice in eppendorf tubes and consisted of 3 μl of assay cocktail (made fresh), 5 μl of 50 ng/μl pTREruΔ53 plasmid (ddH_2_O for the "no DNA" control), 2.5 μl of 2X BC100, and 5 μl of a concentrated mix of GTFs and Pol II that contained 100 ng Pol II, 40 ng TBP, 40 ng TFIIB, 12 ng TFIIEα, 6 ng TFIIEβ, and 60 ng TFIIF in BC100 (BC100 alone was added for the “no protein” control). This mixture was incubated for 50 min at 30°C. Crosslinking nucleotide analog (0.75 μl of 10 mM) was added and the reaction was incubated at room temperature for 2 min. The eppendorf tube lids were opened and the reactions were exposed to a handheld UV (302 nm) device for 2 min, and an additional 30 s on a gel imaging table, also at 302 nm. Next, 0.35 μl of 20 μM CTP and 0.35 μl α-^32^P-CTP (3000Ci/ml) were added. The reactions were mixed by gentle stirring with a pipet tip and incubated at 30°C for 50 min. To improve the signal to background ratio, unincorporated nucleotide was removed by spinning the reactions through a 180 μl bed volume of G-50 fine Sephadex (GE Life Sciences) in Micro Bio-Spin columns (Bio-Rad). The Sephadex was equilibrated with 20 mM Tris-Cl, pH 7.9, 4 mM MgCl_2_, 60 mM KCl, 10 mM DTT, 0.1 mM PMSF, 12% glycerol, 0.1% NP-40. The reactions were run out on 4–20% Tris-Glycine PAGE gels (Novex) at 100 volts. The portion of the gel below 10 kDa was removed prior to drying at 55°C for 1 h. Gels were imaged using BioMax MS film (Kodak) and the corresponding BioMax MS intensifying screens after exposure at −80°C for varying times.

## Results

### A Minimal Transcription System for Pol II Initiation

Our general strategy was to use super-selective crosslinking and labeling techniques to define the functional topography of the Pol II active center, similar to what has previously been described for prokaryotic systems [11, 49]. This entails first assembling functional PICs in the presence of a promoter-containing template, then using initiating substrate analogs to crosslink moieties near the active site of transcription, followed by elongation of the crosslinked substrate residue by the next radioactive NTP. Due to the complexity of the eukaryotic transcription machinery (Pol II and the six GTFs are composed of more than 40 polypeptides), we aimed to simplify the analysis by focusing on a minimal transcription system. Previously, it has been shown that in addition to Pol II, TBP, TFIIB, and TFIIF can suffice for basal (activator-independent) transcription from a supercoiled template containing the Ad ML core promoter [50–53]. TFIIE further stimulates this activity [50, 53]. TFIIH (carrying an ATP-dependent helicase activity) was additionally required for transcription from linear templates, presumably for promoter escape, and it was dispensable for synthesis of the first phosphodiester bond in abortive initiation assays utilizing a dinucleotide primer [54]. However, there is not a general agreement on the dispensability of TFIIH for abortive initiation since it may be dependent on specific experimental conditions (e.g., relative concentrations of GTFs in the reaction), as others have reported a requirement for TFIIH in abortive initiation [55].

**Fig 1:**
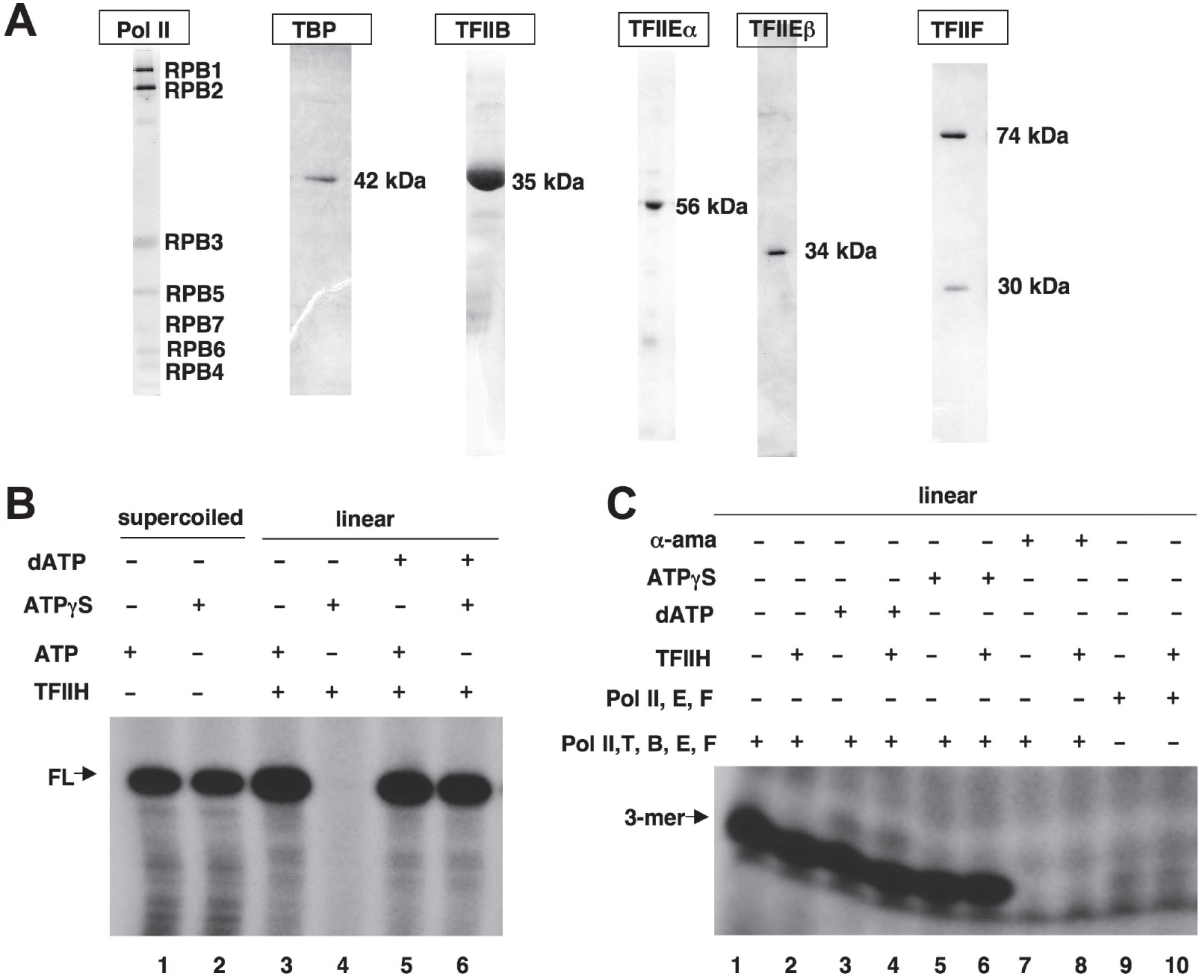
A minimal transcription system sufficient for initiation at the Ad ML promoter. A. SDS-PAGE analyses of Pol II and GTFs used in the study. TFIIE subunits (α and β) were expressed and added to reactions separately. TFIIF was reconstituted prior to use. The Pol II preparation was stained with silver; all others with Coomassie. B. All transcription reactions contained Pol II, TBP, TFIIB, TFIIE, and TFIIF; TFIIH and other additions were as indicated. Following standard transcription reactions with templates with the indicated topologies (supercoiled or linearized), full-length (FL) transcripts were processed for electrophoresis on 4% PAGE containing urea. C. Abortive initiation reactions were primed with the CpA dinucleotide and reactions also contained α^32^P-CTP. TBP and TFIIB were omitted from control reactions in lanes 9 and 10. TFIIH was added as indicated; other additions were also as indicated. α-ama, α-amanitin.

We therefore first ascertained that a system reconstituted from our own homogeneous preparations of Pol II, TBP, TFIIB, TFIIE, and TFIIF sufficed for basal transcription from the Ad ML promoter. For this purpose, transcription factors TBP, TFIIB, TFIIE, and TFIIF were expressed recombinantly in *E*. *coli* and purified; Pol II was purified from a Hela cell line expressing Flag-tagged RPB9 (Fig. 1A, Methods). In standard transcription assays in which steady-state production of a full-length transcript (ca 300 nucleotides) was monitored, Pol II, TBP, TFIIB, TFIIE, and TFIIF sufficed to support efficient transcription from a supercoiled template bearing the Ad ML (Fig. 1B, lane 1). Consistent with its lack of dependence on TFIIH, this system displayed no energy dependence, as ATP could be substituted for the non-hydrolyzable analogue ATPγS (Fig. 1B, lane 2 vs. lane 1). However, as expected, transcription from the same template upon linearization by restriction digestion was completely dependent on energy from ATP (presumably in a TFIIH-dependent fashion), as it was completely abolished when ATPγS was used but was restored when dATP, which cannot be incorporated into RNA, was added back as the energy source (Fig. 1B, lanes 3–6).

**Fig 2:**
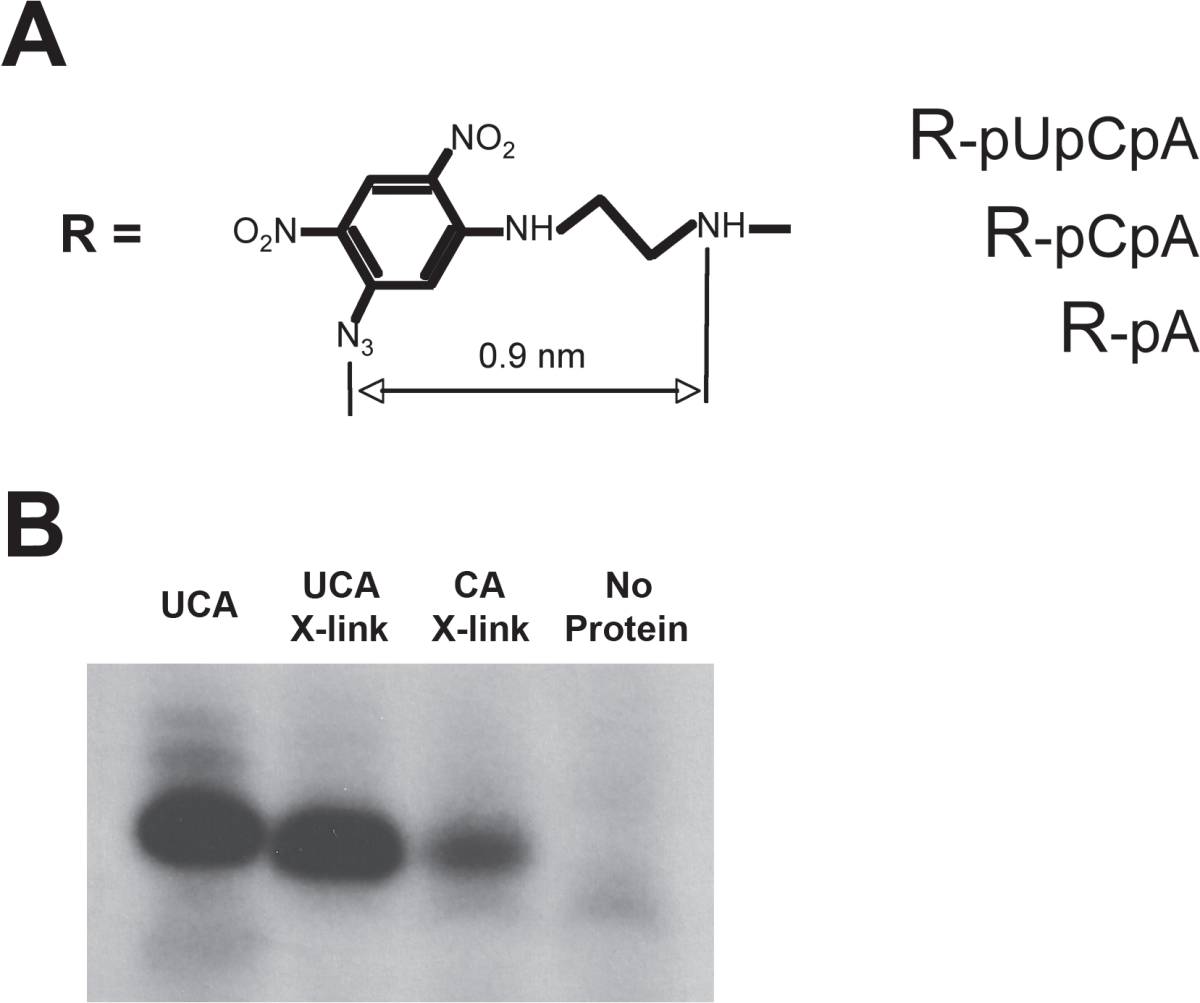
Abortive initiation using crosslinking nucleotide analog substrates. A. Structure of the photoactivatable crosslinking nucleotide analog substrates used in the present study. B. Abortive initiation reactions using a minimal transcription system containing the general transcription factors TFIIB, TFIIE, TFIIF, and TBP and Pol II, and the crosslinking nucleotide analogs as substrates. These reactions were carried out without photo-crosslinking. Left to right: Lane 1—UCA initiating nucleotide. Lane 2—UCA crosslinking analog. Lane 3—CA crosslinking analog. Lane 4—no protein control.

We next analyzed the initiation characteristics of this minimal transcription system. Unlike full-length transcription from a linear template (Fig. 1B), the assay system reconstituted with Pol II, TBP, TFIIB, TFIIE, and TFIIF readily supported formation of a trimer in the presence of the CpA dinucleotide primer (corresponding to the −1/+1 bases around the Ad ML TSS; see also Fig. 2B) and ^32^P-CTP (+2 base) (Fig. 1C, lane 1) from the same template. The abortive production of this trimer (CpApC) was TFIIH-independent (Fig. 1C, lane 2 vs. lane 1) and therefore also energy-independent (lanes 3–6). This product was specifically produced by a Pol II PIC, as it was sensitive to α-amanitin (Fig. 1C, lanes 7 and 8) and was dependent on the key promoter recognition GTFs, TBP and TFIIB (Fig. 1C, lanes 9 and 10).

Thus, we conclude that a minimal system consisting of Pol II, TBP, TFIIB, TFIIE, and TFIIF readily supports formation of the initial phosphodiester bond in abortive transcription assays on a linear Ad ML promoter template. In this regard, our data closely recapitulate the earlier results of Goodrich and Tjian, who were the first to conclude that while TFIIH is required for promoter escape, it is dispensable for early initiation events [54]. These results thus establish the conditions ideally suited for the crosslinking studies described below.

### Crosslinking of TFIIB to an Initiating Nucleotide

Our crosslinking strategy for identifying GTFs located in the active center of Pol II is similar to the activity-dependent affinity labeling described by Grachev et al. for *E*. *coli* RNA Polymerase [49]. In the presence of α-^32^P-CTP (corresponding to the +1 position of the Ad ML promoter) only nucleotide analogs crosslinked in the proper orientation near the active center are elongated (and thus radioactively labeled) by the catalytic activity of Pol II. Crosslinks to other sites on Pol II (specific or not) are not radioactively labeled and are therefore silent, reducing the non-specific background and assuring detection of only the desired crosslinked products. In the present experiments, our aromatic azide photoactivatable analogs (Fig. 2A) can follow two reaction pathways when irradiated with 302 nm UV light. Activation starts with the generation of a nitrene biradical from the azido group, which is accompanied by the liberation of nitrogen. Nitrene crosslinks directly to nearby moieties with little or no preference for residue type. This crosslink is thought to occur on the order of milliseconds to seconds. Competitively, nitrene can react in an intramolecular fashion with the adjacent carbon of the dinitrophenyl ring and form a heterocyclic reactive intermediate. This intermediate reacts relatively slowly and has a preference for nucleophilic residues (Cys, Lys, His, etc.).

We next established whether our crosslinking nucleotide analogs were competent as initiating substrates for transcription. In an abortive initiation assay using the minimal transcription system composed of TBP, TFIIB, TFIIE, and TFIIF with Pol II, both the CpA and UpCpA analogs were competent as primers to produce abortive products when supplemented with α-^32^P-CTP (Fig. 2B). Surprisingly, the mononucleotide A analog did not generate an abortive product, even though unmodified ATP did (data not shown). This could be due to the absence of the two phosphates in the analog, which is expected to significantly reduce the Km of the reactive mononucleotide analog.

**Fig 3:**
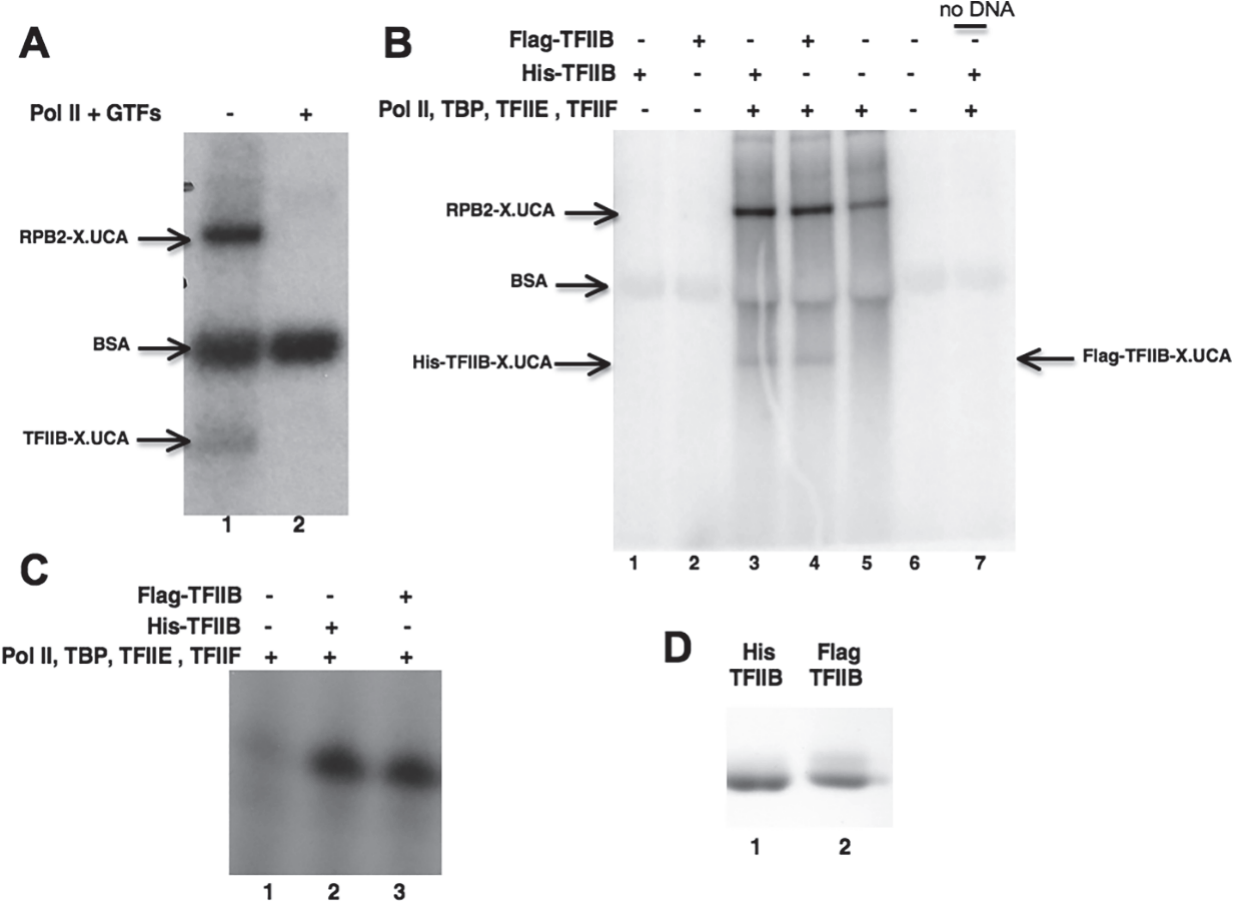
Cross-linking of TFIIB to an Initiating Nucleotide. A. Left to right: Lane 1—Products of a reaction carried out using the trinucleotide crosslinking analog UCA and a complete set of transcription factors (Pol II, TBP, TFIIB, TFIIE, and TFIIF). As indicated, reaction results in the crosslinking of transcription factor TFIIB, as well as the Pol II subunit RPB2. BSA is also labeled, as has been reported previously [56]. Lane 2—control reaction without any transcription factors added (DNA and BSA are present). B. Products of crosslinking reactions as in panel A but with the indicated controls. Left to right: Lane 1 –His-TFIIB only. Lane 2—the same reaction as in lane 1, except that a Flag-tagged version of TFIIB was used. Lane 3—Reaction containing His-tagged TFIIB and complete set of Pol II and general factors (TBP, TFIIE, TFIIF). Lane 4—Reaction as in lane 3, except that Flag-TFIIB was substituted for His-tagged TFIIB. Lane 5—Control reaction containing Pol II, TBP, TFIIE, and TFIIF but no TFIIB. Lanes 6 and 7—control reactions in which, respectively, either the complete set of factors or the template was left out. C. Abortive initiation reactions comparing the activity of the His- and Flag-tagged TFIIB constructs. Left to right: Lane 1—control reaction that contained Pol II and all GTFs except TFIIB. Lane 2—reaction as in lane 1 but supplemented with His-tagged IIB. Lane 3—reaction as in lane 1 but supplemented with Flag-tagged IIB. D. Coomassie stained gel showing the differential electrophoretic mobility of the His and Flag-tagged TFIIB proteins in SDS-PAGE. Left to right: Lane 1—His-TFIIB. Lane 2—Flag-TFIIB.

Using our minimal transcription system, pre-initiation complexes were first assembled on promoter DNA. UV irradiation at 302 nm after incubation with the *UCA X-linker nucleotide analog, followed by extension with α-^32^P-CTP, resulted in two crosslinked, radiolabeled products with molecular weights of approximately 40 kDa and 150 kDa, as visualized by SDS-PAGE and autoradiography (Fig. 3A). The dinucleotide analog failed to produce a radiolabeled product (data not shown). The ∼150 kDa labeled product corresponded to the Pol II subunit Rpb2, consistent with results reported for labeling bacterial β. The mobility of the ∼40 kDa radiolabeled product most closely matched the mobility of TFIIB. We also observed extensive labeling of BSA, a protein component of our transcription assays. In our reaction mixtures BSA is in significant abundance to Pol II and GTFs. The non-specific labeling of BSA by radioactive nucleotides has been reported previously [56].

To confirm the identity of the ca. 40 kDa labeled band as TFIIB, and to ensure that we were not observing a degradation product from another protein species (e.g. Rpb2 or BSA), we carried out additional control experiments (Fig. 3B). First, crosslinking reactions were performed using two differentially tagged TFIIB constructs, one with an N-terminal His6-tag [57] (the version shown in Fig. 2A and used in most of our assays) and one with an N-terminal Flag-tag (sequence DYKDDDDK; [47]). The His6-tag has a molecular mass of approximately 841 Da, while the Flag-tag is approximately 1013 Da. Mass differences of this magnitude are resolvable by high percentage acrylamide gel electrophoresis (Fig. 3D). We first confirmed that our Flag-tagged IIB was competent in an abortive initiation assay (Fig. 3C). Our crosslinking experiments showed that transcription reactions reconstituted from either His-tagged (lane 3) or Flag-tagged TFIIB (lane 4) yield the ca. 40 kDa labeled band. Moreover, the differences in the electrophoretic mobilities of crosslinked Flag- and His6-tagged TFIIBs visualized by autoradiography (Fig. 3B) parallel the differential mobilities seen when the two TFIIB preparations are visualized by Coomassie staining (Fig. 3D). The result in Fig. 3B, which shows the differentially migrating crosslinked Flag- and His6-IIB products, is representative of 5 replicated experiments. Second, we relied on the ability of Pol II to non-specifically initiate at very low levels in the absence of GTFs (see for example Fig. 1C, lane 9 and Fig. 3C, lane 1). We found that when the crosslinking reaction is carried out in the absence of TFIIB, labeling of RPB2 is considerably reduced (Fig. 3B, lane 5), consistent with its compromised ability to form authentic PICs. Importantly, under these conditions, no labeling of the ca. 40 kDa band is detectible (lane 5 versus lanes 3 and 4). Indeed, we find that the labeling of this species is critically dependent on TFIIB concentration (not shown). Other control reactions established that the appearance of the ca. 40 kDa labeled species is dependent on Pol II and the minimal set of GTFs (lanes 1, 2, and 6), as well as on template DNA. Together, these results confirm that the 40 kDa radiolabeled product reflects UCA crosslinking to TFIIB in the context of an authentic PIC.

## Discussion

Here, we have shown that TFIIB is located within ∼9 Å of the −2 site of the template DNA in an active PIC, similar to the position of σ3.2 near the active site of prokaryotic RNAP (11). These results are consistent with structural analyses of Pol II/TFIIB complexes in the absence of promoter DNA [39, 42–44]. Also similar to σ3.2, if the B-reader maintains its position within the active site, its presence will force a termination of the growing RNA chain, resulting in abortive initiation [8, 9, 42]. Indeed, perturbation to TFIIB alters the distribution of abortive transcripts [45].

**Fig 4:**
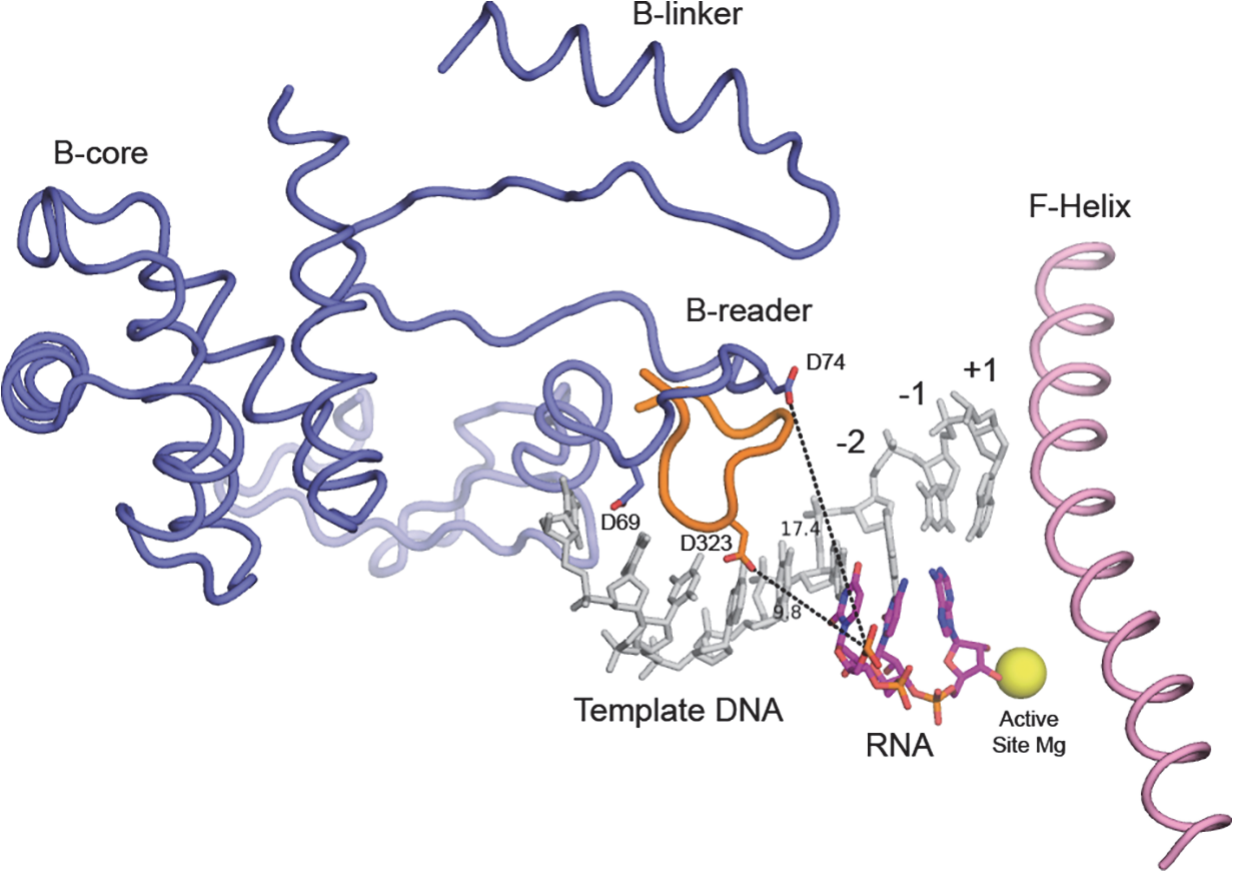
Structural model of the Pol II—TFIIB active site, based on pdb 4BBS (44). The trinucleotide RNA primer (magenta) is representative of the crosslinking reagent used in our studies. The closest TFIIB residue available for reaction with our crosslinker would be D74, 1.7 nm away from the 5' phosphate of the RNA primer. The corresponding position of σ domain 3.2 (orange ribbon) in a bacterial initiation complex is shown. Based on a range of 0.9 nm for the crosslinking moiety, we can speculate that the IIB reader is at least 0.8 nm closer to the active site in an actual initiating complex. Distances are indicated in Å.

Recent structural analyses of Pol II/TFIIB binary complexes reveal a consistent overall architecture [39, 43, 44]. Sainsbury et al. [44] have produced a structural model of the Pol II active center from an initially transcribing Pol II-TFIIB structure containing a promoter based DNA-RNA hybrid. The structure details the positions of the TFIIB-reader elements within the active center. A B-reader strand is positioned to make interactions with the Pol II lid, while the B-reader helix is positioned underneath the lid and adjacent to the −8 position of template DNA [44]. An additional IIB element, the B-linker, is located near the clamp helices. These elements themselves are too far removed from the active site to make contributions to RNA polymerization. A previously disordered region in all other Pol II-TFIIB structures, termed the B-reader loop, is located closest to the active site center. This region connects the B-reader to the B-linker and makes interactions with the growing RNA chain at approximately position −6. Sainsbury et al. have proposed that the B-reader loop is responsible for separating RNA from DNA and directing the RNA chain towards the active site exit tunnel. Excursions to the +1 and −1 sites, according to structure from Sainsbury et al. and the open complex model from Kostrewa et al. appear to be possible [39, 42–44]. This loop is a potential site of crosslinking in our studies. To investigate this idea further, we generated a structural model, based on the most recent TFIIB-Pol II structure from Sainsbury et al., of a Pol II initiating complex containing a 3-residue RNA primer (Fig. 4) [44]. The crosslinking moiety is attached to the 5'-phosphate of the RNA, and would have a range of approximately 0.9 nm for crosslinking to amino acid side chains of TFIIB. The closest TFIIB residue available for crosslinking is approximately 1.7 nm away from the RNA 5'-phosphate, which is greater than the distance coverable by the crosslinking group by 0.8 nm. However, an alignment of σ region 3.2 [9] with the B-reader loop demonstrates that a corresponding aspartate residue in σ is within striking distance of the crosslinker (∼10 nm). We predict that the B-reader loop must adopt a conformation similar to σ3.2 at some point during transcription initiation.

Protein/protein crosslinking suggested physical proximity of TFIIB to TFIIF within the RNAP active site cleft [33]. In our studies, we did not observe crosslinking from any initiating primers to any GTF other than TFIIB, including TFIIF. However, this is a negative result that does not rule out the possibility that TFIIF plays some kind of role within the RNAP active site cleft during transcription initiation.

TFIIF may only be ancillary to transcription, playing a mainly supporting role to TFIIB. Indeed, Cabart et al. [58] have shown recently that, under some conditions, TFIIF is required neither for transcription initiation or promoter clearance. Instead, their results point to a primary supportive role for TFIIF in stabilizing TFIIB within the Pol II complex. The absence of TFIIF in the PIC results in a reduction of transcription at some promoters, however this is due to a destabilizing of TFIIB [58]. These facts do not diminish TFIIF's importance to transcription under some conditions. Mutations in IIB that alter start-site selection can be offset by compensatory mutations in IIF [59, 60]. By controlling the differing positions of the IIB core domain in the PIC and open promoter complexes, TFIIF strongly influences start site selection [61]. In addition, TFIIF may interact with the nontranscribed strand of promoter DNA, fulfilling the role of σ domain 2 [62].

Nature abounds with examples of proteins that share functional and or structural characteristics, yet have no primary sequence in common. TFIIB interacts with promoter DNA both upstream and downstream of the TATA box, similar to σ domains 2 and 4, which contact the −10 and −35 elements, respectively. Like σ, it is the GTF responsible for bringing Pol II (in complex with TFIIF) to the transcription start site. Little to no sequence conservation exists between TFIIB and *E*. *coli* σ70. However, the two proteins do share structural elements responsible for binding DNA. Both contain two helix-turn-helix motifs that serve to bind two DNA elements of promoter DNA, sequences flanking TBP in eukaryotes, and the extended −10 and −35 elements in prokaryotes [63]. In addition, a high degree of structural homology exists between the region connecting the B-linker and B-reader and σ domain's 3 and 4 [39, 42, 43]. Iyer and Aravind speculate that the last unique common ancestor of σ70 and TFIIB recruited RNA Polymerase to specific sites of DNA, to which it was already bound, and thus forged a co-dependent relationship for promoter specific transcription [63].
